# Carnosine enhances actomyosin-ATPase activity under acidic conditions: the role of carnosine in a rigor mortis

**DOI:** 10.1093/jas/skag161

**Published:** 2026-05-18

**Authors:** Toru Hayakawa, Minami Okada, Jun-Ichi Wakamatsu, Haruto Kumura

**Affiliations:** Laboratory of Applied Food Science, Graduate School of Agriculture, Hokkaido University, Research Faculty of Agriculture, Sapporo, Hokkaido 060-8589, Japan; Laboratory of Applied Food Science, Graduate School of Agriculture, Hokkaido University, Research Faculty of Agriculture, Sapporo, Hokkaido 060-8589, Japan; Laboratory of Cell and Tissue Biology, Graduate School of Agriculture, Hokkaido University, Research Faculty of Agriculture, Sapporo, Hokkaido 060-8589, Japan; Laboratory of Applied Food Science, Graduate School of Agriculture, Hokkaido University, Research Faculty of Agriculture, Sapporo, Hokkaido 060-8589, Japan

**Keywords:** imidazole dipeptide, rigor mortis, actomyosin ATPase activity, calcium sensitivity, meat quality

## Abstract

Postmortem changes in skeletal muscle, particularly rigor mortis and the subsequent tenderization process, play a central role in determining meat quality and palatability. Proper progression of rigor mortis is essential, as its disruption leads to severe deterioration in meat texture and eating quality. Although the biochemical mechanisms underlying rigor mortis have been extensively studied, most investigations rely on simplified experimental systems and largely neglect the contribution of endogenous low-molecular-weight compounds present in muscle tissue. Imidazole dipeptides, such as carnosine, are abundant in skeletal muscle and are known to contribute to intracellular homeostasis through antioxidant and metal-chelating properties. Recently, these compounds have also been implicated in the regulation of calcium sensitivity in muscle contraction models. In the present study, we investigated the potential involvement of carnosine in rigor mortis by examining its effects on actomyosin ATPase activity under postmortem-like conditions. Under acidic and low-calcium conditions, which are unfavorable for actomyosin ATP hydrolysis, carnosine significantly enhanced ATPase activity. This enhancement was accompanied by increased tryptophan autofluorescence in the myosin head region upon ATP addition, suggesting an increased affinity between myosin and ATP. These findings indicate that carnosine facilitates the actomyosin ATPase cycle under postmortem conditions by enhancing calcium sensitivity and stabilizing ATP–myosin interactions. Our results suggest that endogenous carnosine contributes to the orderly progression of rigor mortis by promoting ATP depletion and contractile activity in postmortem skeletal muscle, providing new insight into the biochemical factors influencing meat quality and postmortem muscle behavior.

## Graphical abstract

**Graphical Abstract:**
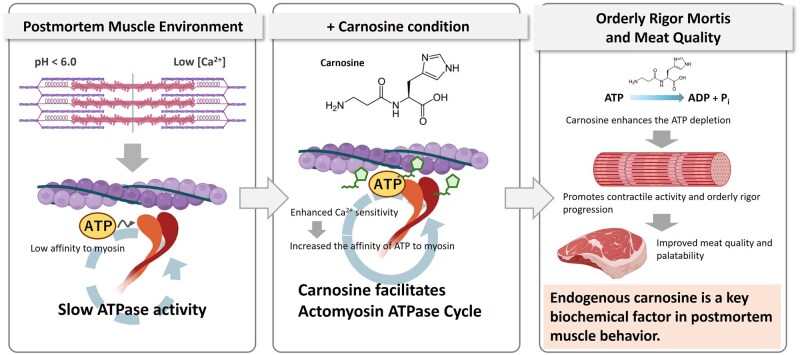

## Teaser

Carnosine, an imidazole dipeptide, enhances the calcium sensitivity of actomyosin and accelerates the actomyosin ATPase cycle under acidic and low-calcium conditions. These findings indicate that endogenous imidazole dipeptides in skeletal muscle actively contribute to the rapid progression of rigor mortis.

## Introduction

Postmortem changes in skeletal muscles, particularly rigor mortis and the subsequent tenderization process, are fundamental phenomena that determine the quality and palatability of meat. The structural organization of muscle fibers, including sarcomere length, myofibrillar proteins, and the integrity of actomyosin complexes, plays a crucial role in meat texture and tenderness (Ertbjerg and Puolanne 2017; Squire 2019). During rigor mortis, ATP depletion and elevated intracellular calcium levels lead to sustained actomyosin cross-bridges, resulting in muscle stiffness. In contrast, the tenderization phase involves the degradation of myofibrillar structures, primarily driven by endogenous proteolytic systems. While the biochemical pathways underlying these processes have been relatively well documented, many studies have relied on simplified models focusing primarily on ionic strength, pH changes, and calcium dynamics.

Recent advances in muscle biochemistry suggest that endogenous small molecules, particularly imidazole dipeptides, may influence postmortem muscle physiology beyond these conventional parameters. Imidazole dipeptides, composed of β-alanine and histidine or methyl histidine, are abundant in skeletal muscle and exhibit a variety of physiological functions, including pH buffering (Abe 2000; Skulachev 2000), antioxidative activity (Boldyrev et al. 1993; Decker et al. 2000), and heavy metal chelator (Baran 2000; Trombley et al. 2000). It has been shown to enhance calcium sensitivity in muscle fibers and contribute to sustained contraction even under low calcium conditions (Dutka et al. 2012). Moreover, carnosine has been implicated in the regulation of enzymatic activities, suggesting its potential role in both muscle contraction and proteolytic processes (Liao et al. 2014). Therefore, understanding the multifactorial regulation of postmortem muscle behavior—including the influence of carnosine—is essential for both meat science and fundamental muscle physiology.

Despite considerable progress in understanding the biochemical and structural basis of postmortem muscle transformation, significant gaps remain, particularly regarding the roles of endogenous dipeptides and small organic molecules. Most studies on rigor mortis and tenderization mechanisms have employed simplified in vitro models, emphasizing changes in pH, calcium ion concentration, and ionic strength, while largely overlooking the potential modulatory effects of physiologically abundant small molecules. Although imidazole dipeptides have been extensively studied for their antioxidative and pH-buffering properties, its direct involvement in postmortem skeletal muscle processes remains poorly characterized. The direct evidence suggesting carnosine’s influence on calcium sensitivity in muscle fibers has been reported by Dutka and Lamb (2004) and Dutka et al. (2012), and they showed increased Ca^2+^ sensitivity without affecting maximal force by using mechanically skinned muscle models. From these reports, we conceived that carnosine affects the ATPase activity of myosin.

The objective of this study is to investigate the physiological role of imidazole dipeptides in postmortem skeletal muscle, focusing specifically on its potential impact on rigor mortis. Notably, one of the most significant biochemical changes in postmortem muscle is a gradual decline in intracellular pH, driven by the net accumulation of protons resulting from continuous ATP hydrolysis (Robergs et al. 2004). This acidic environment alters calcium handling and ATPase activity, thereby influencing muscle contraction dynamics. Although carnosine is known to act as an intracellular pH buffer and modulator of calcium sensitivity, its effects under such low-pH conditions remain poorly understood. We hypothesize that carnosine contributes to the regulation of postmortem muscle contraction through pH-dependent modulation of actomyosin ATPase activity and the calcium responsiveness of the contractile apparatus. To assess this hypothesis, we employed a series of in vitro assays using actomyosin, evaluating ATPase activity and contractile responses under acidic pH conditions, both with and without physiological concentrations of carnosine. This experimental design enables a direct assessment of carnosine’s functional role under conditions that closely simulate the biochemical environment of postmortem muscle. By focusing on the interaction between pH and carnosine, this study aims to clarify a previously underexplored mechanism contributing to the development of rigor mortis.

## Materials and methods

### Preparation of proteins

All procedures conducted during the preparation of proteins were approved by the Hokkaido University Animal Welfare Committee (17-0118). All proteins were prepared from the chicken pectoral muscle, which is composed predominantly of type IIb fibers, in order to minimize the influence of muscle fiber type composition on the experimental results. Furthermore, the pectoral muscle was excised immediately after slaughter and subjected to protein preparation. The time from slaughter to the start of the extraction procedure was less than 30 min for all preparations. Actomyosin was prepared according to the method of Szent-Györgyi (1951). Briefly, minced muscle was extracted in the Weber-Edsall solution (0.6 M KCl, 0.04 M NaHCO_3_, 0.01 M Na_2_CO_3_), and actomyosin fraction was obtained by dilution, centrifugation, and dialysis against 0.6 M KCl. The subfragment of the myosin head region, heavy meromyosin (HMM), was prepared from myosin by α-chymotrypsin digestion based on the method of Margossian and Lowey (1982). The reaction was terminated with phenylmethanesulfonyl fluoride, and crude HMM was further purified by DEAE-TOYOPEARL 650M column chromatography. The fraction containing HMM was eluted in 125 mM KCl solution. The F-actin preparation was prepared from acetone powder according to the method of Pardee and Spudich (1982) with minor modifications. Briefly, acetone powder was suspended in 1 mM ATP and 0.5 mM DTT and centrifuged at 80,000 G for 30 min. The supernatant was suspended at 0.6 M KCl and 1 mM MgCl_2_. After centrifugation at 80,000 × *g* for 3 h, the precipitation was dissolved in 50 mM KCl, 0.02% NaN_3_ and 2 mM Tris–HCl pH 7.5. The solution was stored on ice until use. All of proteins were identified by SDS–polyacrylamide gel electrophoresis (SDS–PAGE) and used for subsequent experiments.

All proteins were prepared from individual chickens and used in the experiments. For each experiment, three technical replicates were performed from the proteins prepared from a single chicken, and the mean value was calculated from three biological replicates obtained from three chickens. A total of 16 chickens were used in this study.

### ATPase activity assay

Actomyosin solution (1.0 mg/mL actomyosin, 150 mM KCl, 1 mM MgCl_2_, 10 µM or 10 nM CaCl_2_, 10 mM carnosine, or MES-NaOH, pH 7.5 or 5.5) was pre-incubated at 25 °C and added ATP solution (final concentration 1 mM) to initiate the reaction. For termination of reaction, 10% trichloroacetic acid was added at defined time points. Inorganic phosphate released during ATP hydrolysis was quantified using the Fiske–Subbarow method (Fiske and Subbarow 1925). The specific activity was expressed as the amount of phosphate released per minute per milligram of actomyosin. Although the reaction time was set at 10 min, preliminary experiments under condition of pH 7.0 and pCa 9 showed instances where the phosphate concentration reached a plateau. Therefore, under this condition, the evaluation was based on a 5-min reaction.

### Superprecipitation of actomyosin

Superprecipitation of actomyosin was examined in a reaction mixture containing 3.0 mg/mL actomyosin, 150 mM KCl and either 10 µM or 1 nM CaCl_2_ at pH 7.0 or 5.5, with 10 mM carnosine or MES-NaOH. The reaction was conducted at 25 °C and initiated by addition of ATP solution (final concentration 1 mM). The formation of superprecipitation was visually observed. For the turbidity measurement, actomyosin was adjusted to 0.7 mg/mL under the same conditions, and changes in turbidity after ATP addition were monitored by measuring absorbance at 660 nm.

### Co-sedimentation assay

The binding of HMM to actin was assessed by a co-sedimentation assay based on the methods of Heier et al. (2017) and Ito et al. (2018). HMM (0.5 mg/mL) and F-actin (0.2 mg/mL) were mixed in a solution of 150 mM KCl, 2 mM MgCl_2_, 1 mM DTT, and either 10 µM or 1 nM CaCl_2_ at pH 7.0 or 5.5, with 10 mM carnosine or MES-NaOH. After incubation at 25 °C, ATP solution (final concentration 2 mM) was added, and samples were centrifuged at 100,000 × *g* for 1 h. The resulting precipitates were subjected to SDS–PAGE according to Laemmli (1970). The relative amount of HMM bound to actin was estimated as the ratio of HMM heavy chain band density to the actin band density using Image J software.

### Tryptophan autofluorescence

The intrinsic tryptophan fluorescence of HMM was measured according to the method of Werber et al. (1972). HMM (0.05 mg/mL) was incubated at 25 °C in a solution containing 150 mM KCl, 1 mM MgCl2, and either 10 µM or 1 nM CaCl_2_ at pH 7.0 or 5.5, with 10 mM carnosine or MES-NaOH. After the addition of ATP (final concentration 0.2 mM), fluorescence spectra were recorded with an excitation at 295 nm and emission from 310 to 400 nm. Background fluorescence from buffer solutions was subtracted, and changes in fluorescence were expressed as the relative increase in maximum intensity after ATP addition. From the obtained spectra, the increased maximum intensity by ATP addition and the relative increase in maximum intensity by ATP addition were calculated. The increased maximum intensity was calculated by subtracting the maximum fluorescence intensity without ATP from that after ATP addition. The relative increase in maximum intensity was calculated as the ratio of the increased maximum intensity by ATP addition to the maximum intensity without ATP.

### Statistical analysis

Data are presented as the means ± standard error (SE). Normality of the data was assessed using the Shapiro–Wilk test, and all datasets were confirmed to follow a normal distribution. Comparison between two groups evaluated using Student’s *t*-test. For multiple-group comparisons, one-way analysis of variance (ANOVA) followed by Tukey’s post hoc test was applied. The significance level was set at *P* < 0.05, and multiple comparisons were corrected using Tukey’s test to control Type I error. Sample sizes were determined based on preliminary experiments using similar experimental systems and were considered sufficient to detect biologically relevant differences. ATPase activity data were analyzed using three-way ANOVA with pH, pCa, and carnosine treatment as factors, followed by Tukey’s post hoc test. Statistical analyses were performed using JMP Pro software version 17.0.0 (SAS Institute Inc., Cary, NC, USA).

## Results

### ATPase activity of actomyosin

**Figure 1:**
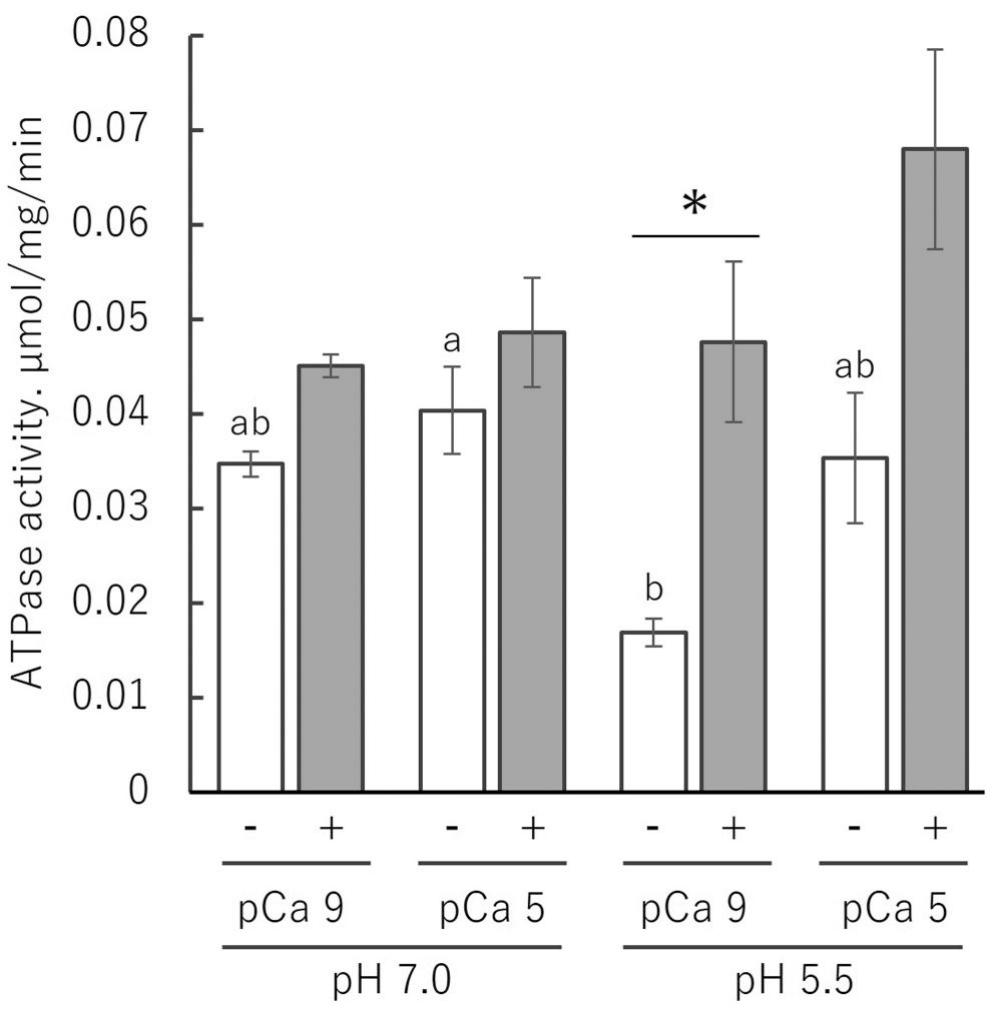
Effect of carnosine on actomyosin ATPase activity. ATPase activity was determined under the neutral or acidic conditions with or without 10 mM carnosine. Calcium concentrations were pCa 9 or 5. ATPase activity was calculated from free phosphate amount per protein 1 g and 1 min. Opened circles and bars: without carnosine; closed circles and bars: with 10 mM carnosine. Error bars: SE. *P < 0.05; a, b, c: different letters indicate the significant difference within the group of the absence of carnosine (*P* < 0.05). Three-way ANOVA revealed a significant interaction between pH and pCa (*P* = 0.012), whereas no interaction was observed between pCa and carnosine (*P* = 0.994). *n* = 3, as biological replicates.

To evaluate the efficiency of carnosine, we determined the ATP hydrolysis activity of actomyosin under various pH and Ca^2+^ concentration conditions (Figure 1). In the absence of carnosine, the activity at pH 5.5 was lower than that at pH 7.0, and this decrease was particularly pronounced under low Ca^2+^ conditions. In contrast, in the presence of carnosine, high activity was observed at pH 5.5 and pCa 5; however, no significant differences were detected among the four conditions tested. The addition of carnosine increased the activity under all conditions, but a significant increase was observed only at pH 5.5 and pCa 9. Three-way ANOVA (pH × pCa × carnosine) revealed a significant interaction between pH and carnosine (*P* < 0.012), indicating that the enhancing effect of carnosine was greater under low pH conditions. No significant interaction was found between carnosine and pCa. These results suggest that carnosine enhances the ATP hydrolysis activity of actomyosin under acidic conditions.

### Superprecipitation of actomyosin

**Figure 2:**
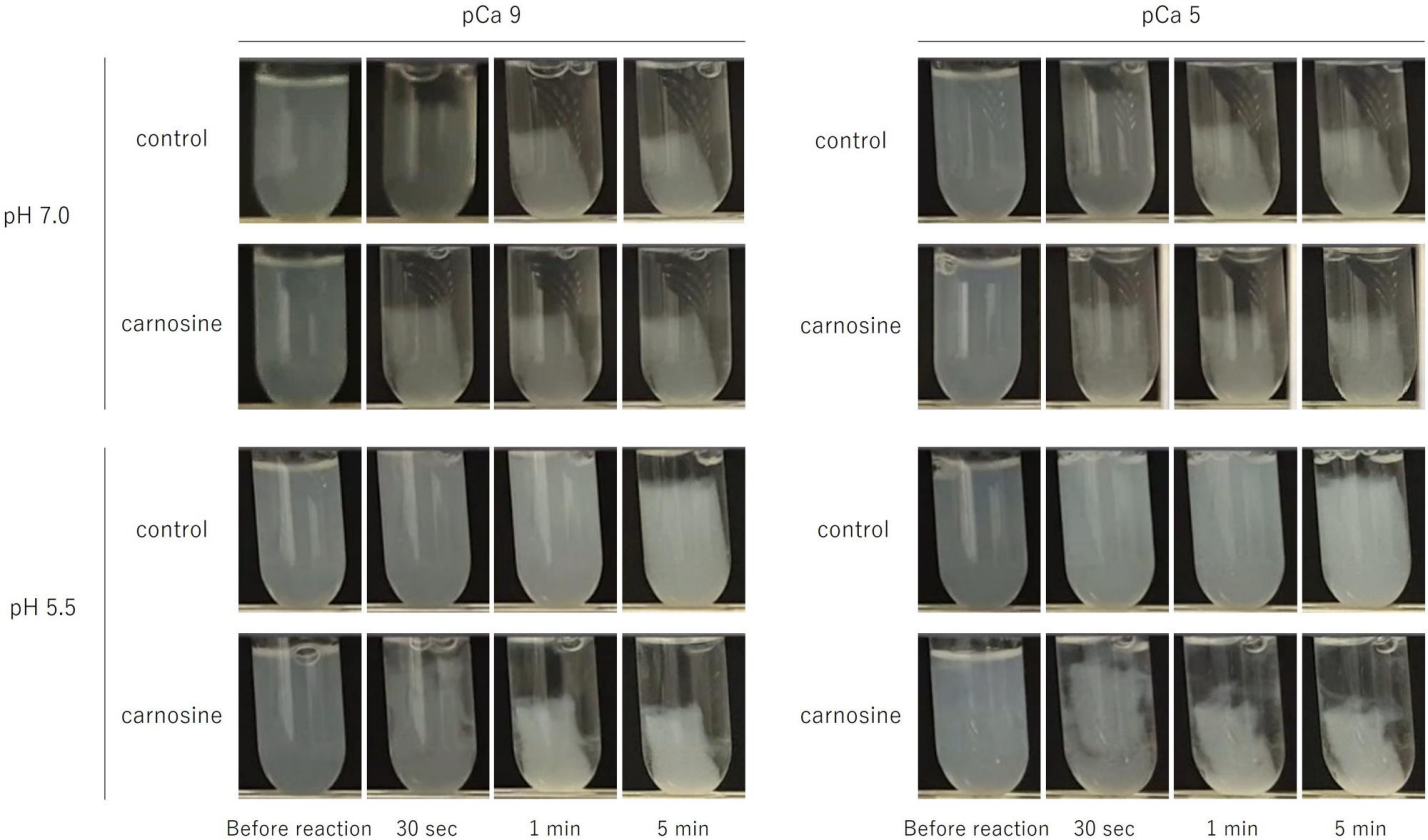
Effect of carnosine on actomyosin superprecipitation. ATP solution (final conc., 1 mM) was added to actomyosin suspension (3.0 mg/mL in 150 mM KCl with or without 10 mM carnosine) under the neutral or acidic condition. The appearance of test tubes was observed.

**Figure 3:**
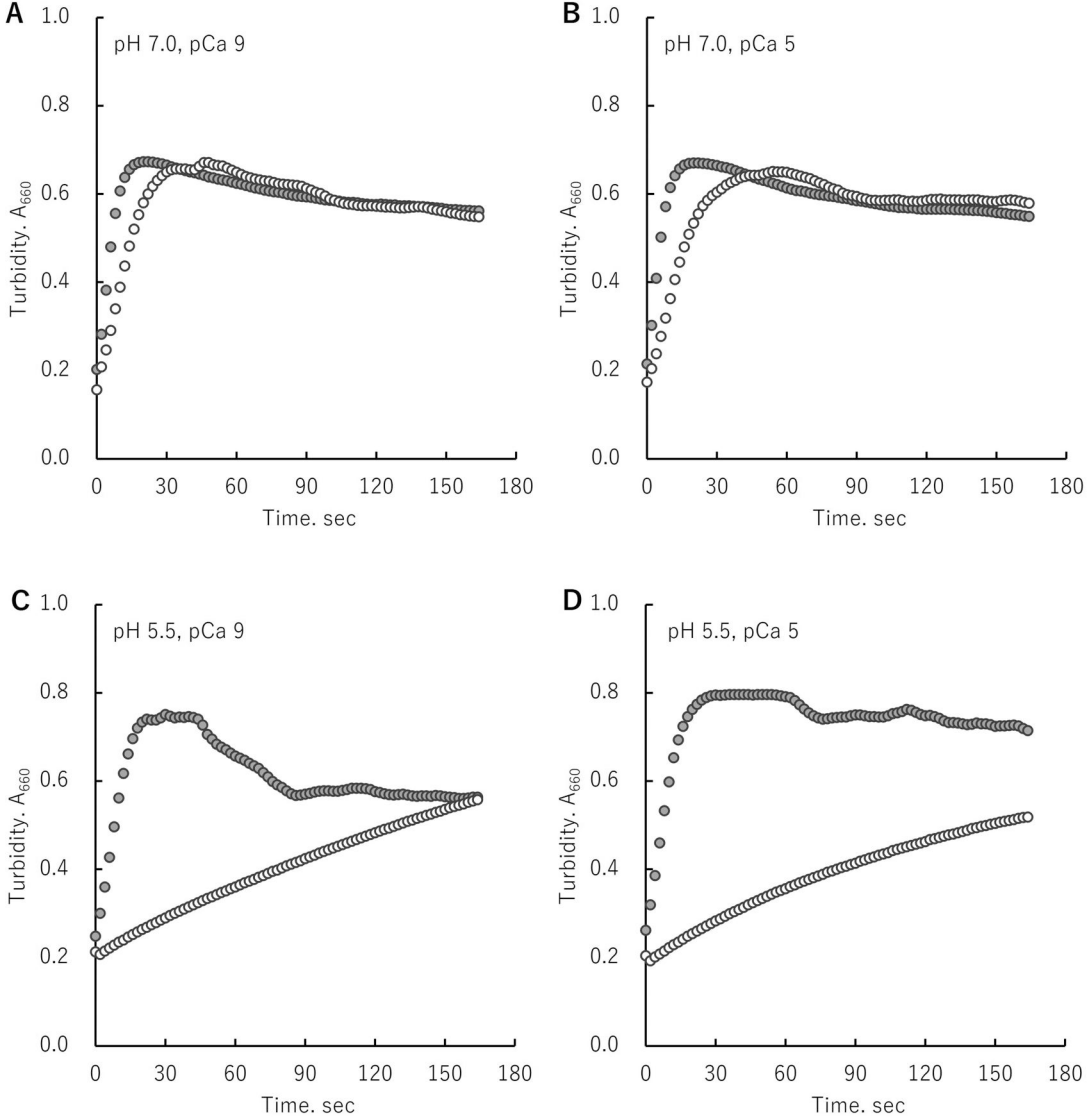
Effect of carnosine on the turbidity of actomyosin superprecipitation. ATP solution (final concentration, 1 mM) was added to actomyosin suspension (0.7 mg/mL in 150 mM KCl with or without 10 mM carnosine) under the neutral or acidic conditions. The turbidity was determined by measuring absorbance at 660 nm. Opened circles: without carnosine; closed circles: with 10 mM carnosine. Three biological replicates were performed, and representative data were shown. (A) pH 7.0, pCa 9. (B) pH 7.0, pCa 5. (C) pH 5.5, pCa 9. (D) pH 5.5, pCa 5.

To investigate the impact of carnosine on actomyosin contractility, we observed the superprecipitation of actomyosin in the presence of carnosine (Figure 2). After the addition of ATP at neutral pH, actomyosin shrank and formed precipitation. The precipitation continued to shrink 30 s after the addition of ATP and maintained its form at 5 min after the addition of ATP. The changes in the appearance of actomyosin solutions at neutral pH were similar between pCa values of 9 and 5, in the presence and absence of carnosine. However, at a pH of 5.5, regardless of pCa, the formation of actomyosin precipitates upon the addition of ATP was much slower in the absence of carnosine. This was due to the inability of actomyosin to hydrolyze ATP at acidic pH. However, in the presence of carnosine, actomyosin precipitated upon addition of ATP regardless of Ca^2+^ concentration. The actomyosin precipitation shrank 5 min after ATP was added. To evaluate the contractile properties of actomyosin in the presence of carnosine, we measured the turbidity changes of the actomyosin solution upon addition of ATP (Figure 3). The turbidity of the actomyosin solution sharply increased upon addition of ATP at neutral pH (Figure 3A and B). The times to reach the highest turbidity in the absence of carnosine were 46 and 54 s at pCa of 9 and 5, respectively. In contrast, the times to reach the highest turbidity in the presence of carnosine were 20 and 22 s at pCa of 9 and 5, respectively. The reactivity of actomyosin to ATP was observed to be higher in the presence of carnosine than in its absence. At an acidic pH of 5.5, the turbidity of the actomyosin solution gradually increased in the absence of carnosine, while in the presence of carnosine, it increased rapidly within 25 s of adding ATP (Figure 3C and D). The increase in turbidity in the presence of carnosine occurred irrespective of the concentration of Ca^2+^. Therefore, carnosine could be suggested to activate the ATP hydrolysis cycle under acidic pH conditions.

### Co-sedimentation of actin and HMM

**Figure 4:**
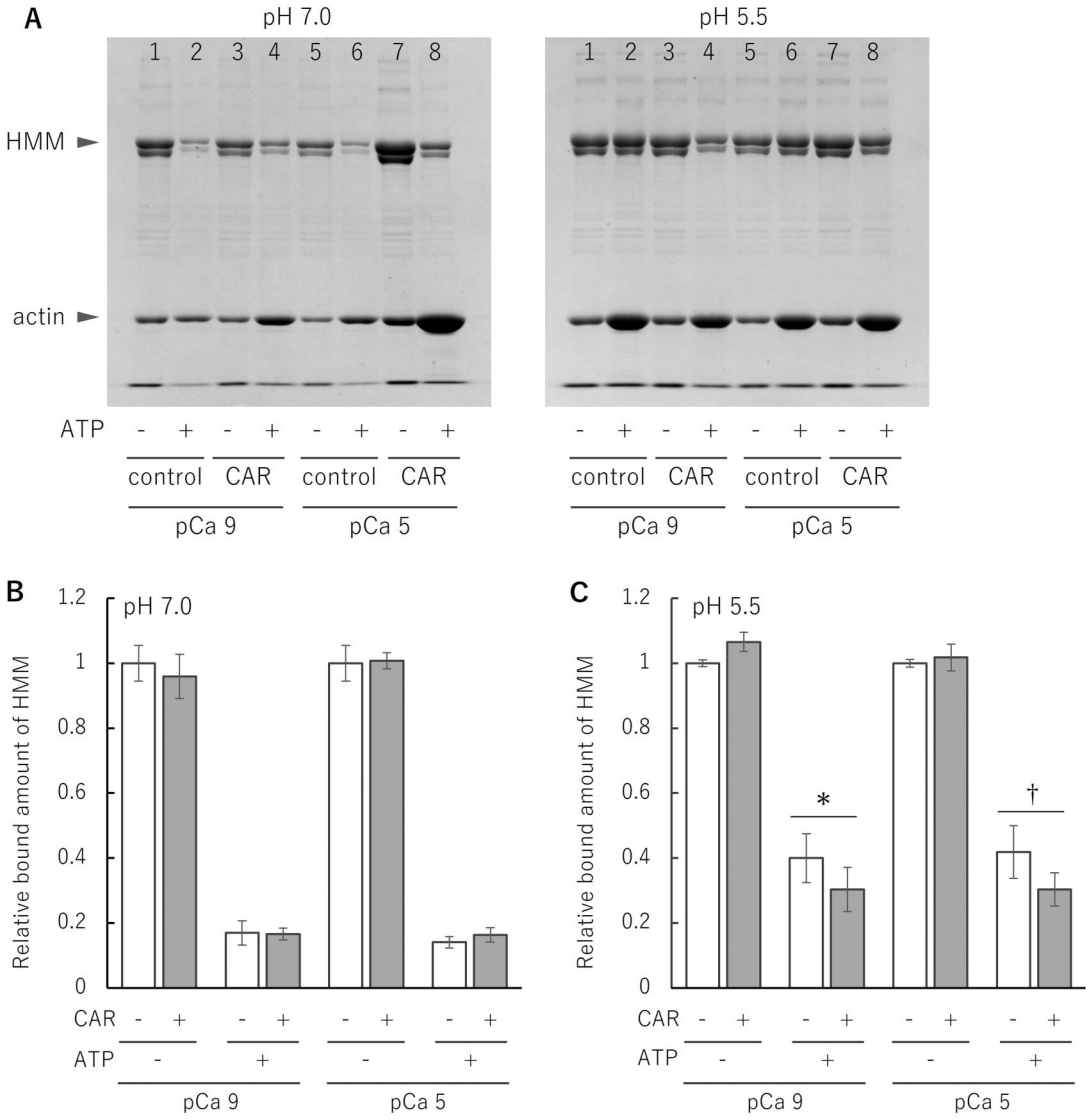
Effect of carnosine on the co-sedimentation between HMM and actin. HMM–actin mixture, containing 0.5 mg/mL of HMM and 0.2 mg/mL of actin in 150 mM KCl, pH 7.0 or 5.5, was incubated at 25 °C for 10 min, followed by the addition of ATP (final concentration: 1 mM). After centrifugation, precipitation was collected and dissolved in 0.5 M NaCl. The solution was subjected to SDS–PAGE, and the band density of HMM was determined by densitometry. (A) SDS–PAGE image of co-sedimentation assay. (B) Relative amount of HMM bound to actin at pH 7.0. (C) Relative amount of HMM bound to actin at pH 5.5. The relative amount of HMM bound to actin was estimated from the ratio of the HMM heavy chain band density to the actin band density in the pellet fraction after co-sedimentation, and expressed relative to the corresponding value obtained on the control condition (in the absence of carnosine and ATP) at each pH and pCa. Opened bars: without carnosine; closed bars: with 10 mM carnosine. Error bars: SE. *P < 0.05; †: *P* < 0.10. *n* = 3, as biological replicates.

To investigate why carnosine enhances actomyosin ATP hydrolytic activity under acidic conditions, we focused on the ATPase cycle. During actomyosin ATP hydrolysis, dissociation between myosin and actin occurs (Okami et al. 2020). We evaluated the effect of the presence of carnosine on the binding between the myosin head domain and actin using a co-sedimentation assay with HMM and actin (Figure 4).

At neutral pH, the intensity of the HMM heavy chain band decreased upon ATP addition under all conditions (Figure 4A). This indicates that ATP addition caused HMM molecules to dissociate from the actin–HMM complex. The band densities of the HMM heavy chain and actin were quantified by densitometric analysis of SDS-PAGE to calculate the amount of HMM bound to actin. The results showed that at neutral pH, the amount of HMM bound to actin was consistently reduced by ATP addition, regardless of Ca^2+^ concentration or the presence of carnosine (Figure 4B). These findings indicate that within the neutral pH range, carnosine does not affect the dissociation of the actomyosin complex associated with ATP hydrolysis.

At acidic pH, ATP addition also decreased the amount of HMM bound to actin (Figure 4A). In contrast, in the presence of carnosine, the amount of HMM bound to actin upon ATP addition was lower than that observed in the absence of carnosine (Figure 4C). This suggests that under acidic conditions, the presence of carnosine enhances the dissociation of HMM molecules from the actin–HMM complex following ATP addition. Collectively, these results indicate that carnosine modulates the ATP hydrolysis cycle of the actomyosin complex under acidic conditions and promotes ATP hydrolysis by facilitating the release of myosin heads from actin filaments.

### Conformational change of HMM by ATP addition

**Figure 5:**
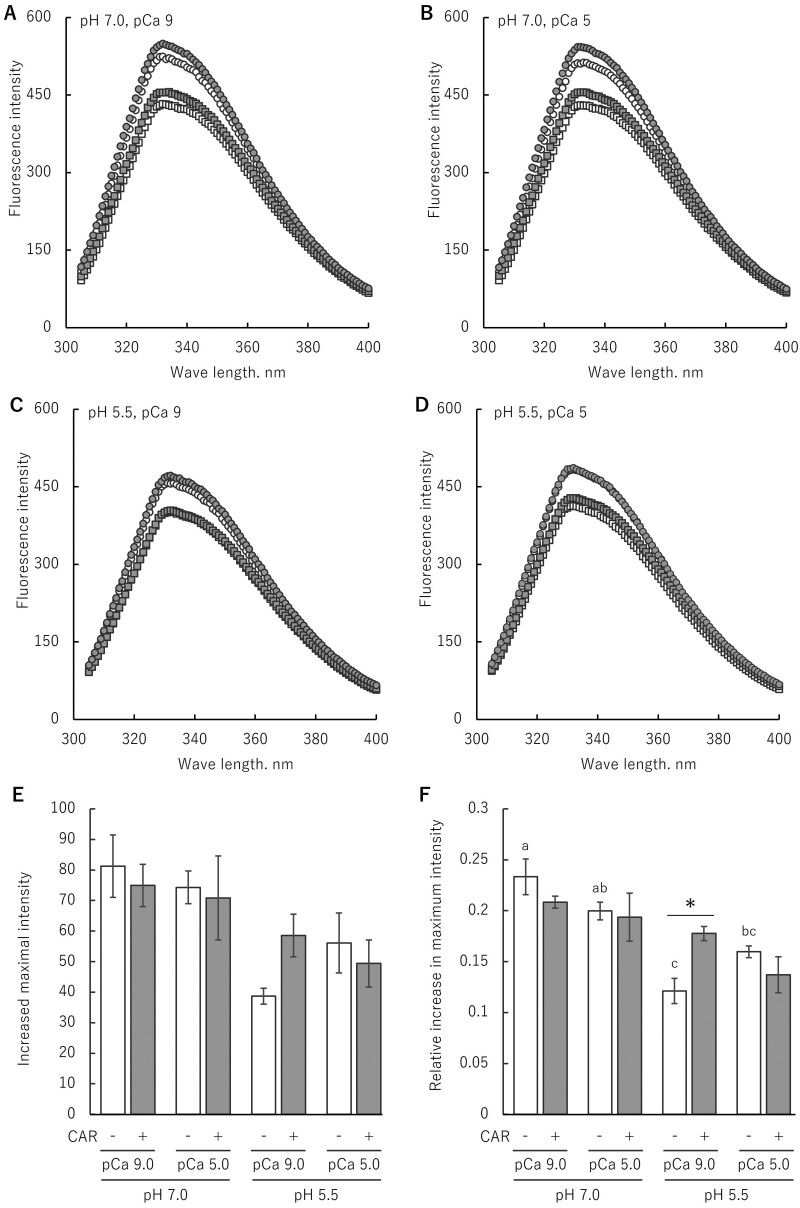
Effect of carnosine on the tryptophan autofluorescence of HMM. HMM mixture (0.05 mg/mL HMM, 150 mM KCl, 1 mM MgCl_2_, with or without 10 mM carnosine) was incubated at 25 °C for 10 min. After addition of ATP (final concentration: 0.2 mM), fluorescence spectra were obtained with an excitation wavelength of 295 nm and emission wavelengths ranging from 310 to 400 nm. (A)–(D) fluorescence spectra before and after the addition of ATP. Opened symbols: without carnosine; closed symbols: with 10 mM carnosine; circles: with ATP; squares: without ATP. Representative data was shown. (A) pH 7.0, pCa 9. (B) pH 7.0, pCa 5. (C) pH 5.5, pCa 9. (D) pH 5.5, pCa 5. (E) Increased maximum intensity by the addition of ATP. The data were represented as the difference between the maximum fluorescence intensity obtained from HMM spectra with added ATP and those without ATP. (F) Relative increase in maximum intensity by the addition of ATP. The data were represented as the value of the increased maximum fluorescence intensity by the addition of ATP, relative to the maximum fluorescence intensity without ATP addition. Opened bars: without carnosine; closed bars: with 10 mM carnosine. Error bars: SE. *P < 0.05. a, b, c: different letters indicate the significant difference within the group of the absence of carnosine (*P* < 0.05). *n* = 3, as biological replicates.

It is well established that ATP binding to the head region of myosin induces a conformational change of myosin, resulting in an increase in intrinsic tryptophan fluorescence (Werber et al. 1972). To investigate the effect of carnosine on ATP binding to the myosin head region, ATP-induced structural changes in HMM were analyzed by monitoring intrinsic tryptophan fluorescence (Figure 5). Under all conditions, the maximum fluorescence emission was observed at 331–333 nm, and no difference in the maximum fluorescence intensity was detected in the presence or absence of carnosine (Figure 5A–D). Therefore, the relative increase in fluorescence intensity upon ATP addition was evaluated (Figure 5E and F). This value at pH 5.5 was lower than that at pH 7.0, suggesting that ATP binding to HMM is suppressed under acidic conditions. Furthermore, whereas no change was observed in the presence of carnosine under neutral conditions, this value increased significantly in the presence of carnosine under acidic conditions at pCa 9 (Figure 5F). These results suggest that carnosine enhances ATP binding to the myosin head region of HMM under acidic conditions with low calcium ion concentrations.

## Discussion

To explore the role of imidazole dipeptides, which are abundant in skeletal muscle, in the development of rigor mortis, we examined their effects on the muscle contraction mechanism under acidic conditions by evaluating actomyosin ATPase activity. Our findings suggest that carnosine may attenuate the decline in actomyosin ATPase activity associated with a reduction in pH (Figure 1). Because myosin ATPase activity is known to decrease when pH deviates from its optimum (Penny 1967; Blanchard et al. 1984), the presence of carnosine may partially rescue this pH-induced loss of activity. In addition, the observation that ATPase activity was not reduced by pH lowering at high calcium ion concentrations implies that the protective effect of carnosine is likely related to modulation of calcium sensitivity. ATPase activity in the presence of carnosine increased by approximately 1.048 ± 0.169-fold at pCa 9 and 1.506 ± 0.329-fold at pCa 5 when pH decreased from 7.0 to 5.5. In contrast, in the absence of carnosine, ATPase activity was reduced to 0.494 ± 0.058-fold at pCa 9 and 0.976 ± 0.300-fold at pCa 5 relative to the value at pH 7.0. These results indicate that the effect of carnosine is markedly enhanced under acidic conditions and is consistent with the significant interaction observed between pH and carnosine.

Although enhancement of myosin ATPase activity by carnosine has previously been reported (Avena and Bowen 1969), the extremely high concentration of carnosine used in that study (0.1 M) makes direct comparison with the present results difficult, and the underlying mechanism was not addressed. To further examine the influence of carnosine on actomyosin ATPase function, we employed actomyosin superprecipitation and the associated turbidity changes as a simplified model of the contractile mechanism (Figures 2 and 3). Under acidic conditions, carnosine consistently promoted actomyosin superprecipitation, supporting the ATPase activity data. Notably, superprecipitation was enhanced by carnosine regardless of calcium ion concentration, in contrast to the ATPase results. This discrepancy may reflect differences in reaction conditions, particularly the presence of magnesium ions, and suggests that interactions between carnosine and divalent metal ions could contribute to the facilitation of superprecipitation. Collectively, these observations imply that carnosine may modulate calcium sensitivity under acidic conditions and thereby alleviate the reduction in ATPase activity.

Caffeine, which shares an imidazole structure with carnosine, has been reported to increase calcium sensitivity in skeletal muscle contraction (Wendt and Stephenson 1983; Lamont and Miller 1992) and to induce structural changes in the skeletal muscle ryanodine receptor (RyR) (des Georges et al. 2016; Murayama et al. 2018). By analogy, carnosine may also influence the structure of ATP-binding regions in actomyosin and myosin. Based on this possibility, we investigated the effects of carnosine on ATP-dependent binding and dissociation between HMM and actin, as well as on ATP-induced structural changes in HMM, using fluorescence spectroscopy and co-sedimentation assays. Under acidic conditions, co-sedimentation analysis revealed that the amount of HMM bound to actin decreased in the presence of carnosine (Figure 4). This finding suggests that, in the presence of carnosine, a larger fraction of HMM molecules may remain in a dissociated state by binding ATP, implying an increased affinity between HMM and ATP.

During the actin-activated ATPase cycle of myosin, the duration of myosin–actin interaction is a key determinant of ATPase activity, with shorter binding times favoring higher activity (Walklate et al. 2016). Accordingly, carnosine may enhance ATPase activity under acidic conditions by increasing the affinity of myosin for ATP and thereby shortening the lifetime of actin-bound states. Previous work has shown that pH reduction does not substantially affect ATP binding to myosin but increases myosin–ADP affinity (Debold et al. 2008), suggesting that pH-induced suppression of ATPase activity may arise from inhibited ADP release from the myosin head region (HMM), leading to prolonged actin binding.

To further assess nucleotide binding to HMM, intrinsic tryptophan fluorescence was measured in the presence of ATP. Under acidic and low calcium ion concentration conditions, the relative fluorescence intensity increased in the presence of carnosine (Figure 5). Because increased fluorescence intensity reflects nucleotide-bound states of HMM (Werber et al. 1972; Ito et al. 2018), and because ADP is known to be rapidly released from the myosin head region when ATPase activity is high (Ito et al. 2009), this result is most consistent with an increase in ATP-bound HMM. In contrast, no carnosine-dependent difference in relative fluorescence intensity was observed under neutral conditions, consistent with the actomyosin ATPase results (Figure 1). Moreover, the maximum fluorescence intensity upon ATP addition was lower under acidic than neutral conditions (428.22 vs. 380.62), indicating that HMM-ATP binding is generally suppressed at low pH. Although Debold et al. (2008) reported no effect of pH on myosin-ATP binding, the strongly acidic conditions used in the present study (pH 5.5) may have been sufficient to influence this interaction. Under low calcium ion concentrations, HMM-ATP binding appeared to be further suppressed, whereas the presence of carnosine partially restored this binding.

An unresolved question is why the effect of carnosine becomes apparent only under acidic and low calcium ion concentration conditions. These conditions are likely unfavorable for actomyosin ATP hydrolysis, thereby making any facilitatory effect of carnosine more evident. In contrast, under neutral pH or high calcium ion concentrations, the contractile system may already operate near optimal efficiency, reducing the apparent need for carnosine-mediated rescue. A plausible mechanism may involve the interaction between the myosin head region and divalent metal ions. Divalent cations play a critical role in substrate binding and regulation of the ATPase cycle (Peyser et al. 1997; Tkachev et al. 2013). When Mg^2+^ is coordinated to the myosin head, release of inorganic phosphate becomes rate-limiting, whereas Ca^2+^ coordination allows more rapid cycle progression (Tkachev et al. 2013). In addition, free Mg^2+^ has been reported to enhance myosin–ADP affinity (Kobayashi et al. 2019). Thus, under low calcium ion conditions, Mg-ATP may predominate, suppressing ADP release and reducing ATPase activity. Under such unfavorable conditions, the ability of carnosine to promote myosin-ATP binding may become more pronounced. Moreover, although the chemical role of carnosine in enhancing actomyosin ATPase activity remains to be clarified, interesting findings were obtained from measurements of tryptophan autofluorescence of HMM. In the presence of carnosine, the maximum fluorescence intensity in the absence of ATP tended to be higher than that observed without carnosine, suggesting a structural change in the HMM molecule induced by carnosine (Figure 5). This suggests that carnosine may enhance ATPase activity by inducing conformational changes in the myosin head region, thereby facilitating ATP binding or ADP release.

Previous studies of rigor mortis have largely relied on simplified in vitro models, leaving certain aspects unresolved. In particular, it has remained unclear why ATP depletion and completion of rigor mortis proceed even under conditions in which pH decreases and ATPase activity would be expected to cease. This difficulty arises in part from the temporal gap between pH reduction and calcium ion accumulation in postmortem skeletal muscle. The present findings suggest that carnosine enhances calcium sensitivity of actomyosin ATPase activity under acidic conditions, providing a plausible explanation for this phenomenon. Moreover, our results imply that imidazole dipeptides, known for their buffering capacity and metal ion chelation properties, may also function as modulators of the skeletal muscle contractile apparatus by enhancing calcium sensitivity. This raises the possibility that imidazole dipeptides contribute not only to postmortem muscle physiology but also to the regulation of skeletal muscle function during exercise-induced environmental changes, highlighting potential relevance to exercise physiology.

In summary, by examining an actomyosin contractile system, we sought to clarify the physiological role of imidazole dipeptides in rigor mortis, with particular emphasis on the effects of carnosine on ATPase activity. Our results suggest that carnosine enhances actomyosin ATPase activity under acidic or low calcium ion conditions and promotes ATP binding to the myosin head region under acidic and low calcium ion concentration conditions. Taken together, these findings support the hypothesis that carnosine accelerates the ATPase cycle by increasing calcium sensitivity of actomyosin under acidic conditions. In postmortem skeletal muscle, this endogenous compound may therefore facilitate rapid ATP depletion and ensure the progression of rigor mortis.

## Abbreviations

DTT: dithiothreitol
HMM: heavy meromyosin
PMSF: phenylmethanesulfonyl fluoride
SDS: sodium dodecyl sulfate
SDS-PAGE: SDS-polyacrylamide gel electrophoresis
TCA: trichloroacetic acid
